# Nanospheres Loaded with Curcumin Improve the Bioactivity of Umbilical Cord Blood-Mesenchymal Stem Cells via c-Src Activation during the Skin Wound Healing Process

**DOI:** 10.3390/cells9061467

**Published:** 2020-06-15

**Authors:** Do-Wan Kim, Chang-Hyung Choi, Jong Pil Park, Sei-Jung Lee

**Affiliations:** 1 Department of Pharmaceutical Engineering, Daegu Haany University, Gyeongsan 38610, Korea; sosr200211@gmail.com; 2 Division of Cosmetic Science and Technology, Daegu Haany University, Gyeongsan 38610, Korea; cchoi@dhu.ac.kr; 3 Department of Food Science and Technology, Chung-Ang University, Anseong 17546, Korea

**Keywords:** curcumin nanospheres, UCB-MSCs, motility, c-Src, wound repair

## Abstract

Curcumin, a hydrophobic polyphenol derived from turmeric, has been used a food additive and as a herbal medicine for the treatment of various diseases, but the clinical application of curcumin is restricted by its poor aqueous solubility and its low permeability and bioavailability levels. In the present study, we investigate the functional role of a nanosphere loaded with curcumin (CN) in the promotion of the motility of human mesenchymal stem cells (MSCs) during the skin wound healing process. CN significantly increased the motility of umbilical cord blood (UCB)-MSCs and showed 10,000-fold greater migration efficacy than curcumin. CN stimulated the phosphorylation of c-Src and protein kinase C which are responsible for the distinctive activation of the MAPKs. Interestingly, CN significantly induced the expression levels of α-actinin-1, profilin-1 and filamentous-actin, as regulated by the phosphorylation of nuclear factor-kappa B during its promotion of cell migration. In a mouse skin excisional wound model, we found that transplantation of UCB-MSCs pre-treated with CN enhanced wound closure, granulation, and re-epithelialization at mouse skin wound sites. These results indicate that CN is a functional agent that promotes the mobilization of UCB-MSCs for cutaneous wound repair.

## 1. Introduction

Stem cell migration, a dynamic biological event involving inflammation, new tissue formation, vascularization, and finally tissue remodeling, is an essential process during the healing of skin wounds [1,2]. Many studies in the area of translational medicine have emphasized that the migration capacity of stem cells activated by specific microenvironments known as niches is necessary for improving the regeneration of injured tissues [3,4,5,6]. To enhance the bioactivity of stem cells, there have been many therapeutic attempts to manipulate stem cell niches and behaviors, especially via using the genetic modifications and through the use of biochemical cocktails [7,8], but these are considered impractical for clinical use due to unintended potential adverse effects. Therefore, the development of safe and effective therapeutic strategies to modulate stem cell migration is a priority in the research on improving the skin wound healing process. 

Curcumin, a natural polyphenol component of turmeric found in *Curcuma longa* (Linn.) is both widely available and inexpensive and has traditionally been linked to wound healing activity [9,10]. It has long been consumed by humans without any apparent adverse reactions [11]. Accumulating evidence has indicated that curcumin possesses pharmacological effects that modulate numerous molecular targets, such as growth factors, reactive oxygen species, cellular factors, transcription factors, and apoptotic genes [12,13]. Recent reports have shown that curcumin exerts protective effects on stem cell proliferation, differentiation, and aging [14]. However, despite the enormous curative potential of curcumin, the clinical applications of curcumin have been restricted by its hydrophobicity, poor gastric absorption rate, photosensitivity, and low bioavailability [15]. In an effort to enhance its bioavailability, we recently developed a nanotechnology-based curcumin delivery system in which curcumin is incorporated into different formulations using nanoparticles and lecithin, a vegetable-based phospholipid that is a major component of all cell membranes [16,17]. This active nanosphere, when loaded with curcumin (designated henceforth as CN), has the ability to improve its aqueous-phase solubility and bioavailability levels, showing many biological functions in vivo and in vitro [16,17]. However, the physiological significance of CN with regard to the guiding of the migratory behavior of stem cells has yet to be characterized.

Human umbilical cord blood-derived mesenchymal stem cells (UCB-MSCs), self-renewing multipotent progenitors, are among the most abundant sources of non-embryonic stem cells [18] and have the capacity to differentiate into multiple cell types with low immunogenicity. They are also free of any ethical controversy [18,19,20]. Thus, human UCB-MSCs can be regarded as the most potential stem cell source, and their use has led to major advances in cell therapy and regeneration strategies in the areas of bone regeneration and spinal cord injuries [21,22]. Given the migration ability of MSCs via circulation to tissue damage sites, many studies have also focused on the development of new molecules which regulate MSC migration during the wound healing, damage repair, and regeneration process [23,24,25,26]. Thus, in this study, we investigated the functional role of CN in promoting the migratory behavior of UCB-MSCs during the wound healing process.

## 2. Materials and Methods

### 2.1. Materials

Human umbilical cord blood-derived mesenchymal stem cells (UCB-MSCs) isolated and expanded as reported previously [20] were kindly provided by Prof. Ho Jae Han (Seoul National University, Korea). The experimental use of UCB-MSCs was approved by the Seoul National University Institutional Review Board (SNUIRB No E1707/002-003) at July 13, 2017. These cells have been characterized to express CD105 (99.6%) and CD73 (96.3%), but not CD34 (0.1%), CD45 (0.2%) and CD14 (0.1%). They were positive for HLA-AB, but generally not for HLA-DR [20]. The UCB-MSCs can be differentiated into various cell types such as osteoblasts, chondrocytes, and adipocytes upon in vitro induction with the appropriate osteogenic, chondrogenic, and adipogenic differentiation stimuli [20]. In present study, all the experiments were carried out with cells from passage 7. *Curcuma Longa* Linn (powdered form), 2-aminoethyldiphenyl borate (DPBA), and lecithin (L-α-phosphatidylcholine) were obtained from Sigma-Aldrich (St. Louis, MO, USA). Organic solvents such as toluene and dichloromethane were purchased from Fisher Scientific (Waltham, MA, USA). Fetal bovine serum (FBS) and phosphate buffered saline (PBS) were purchased from GE Healthcare (Logan, UT, USA). The following antibodies were purchased: F-actin antibody (abcam, Cambridge, MA, USA); c-Src, p-c-Src, pan-PKC, p-PKC, ERK, p-ERK, c-Jun N-terminal kinase (JNK), p-JNK, p38 MAPK, p-p38 MAPK, NF-κBp65, p-NF-κBp65, IκBα, p-IκBα, α-actinin, profilin-1, and β-actin antibodies (Santa Cruz Biotechnology, Paso Robles, CA, USA); horseradish peroxidase (HRP)-conjugated goat anti-rabbit and goat anti-mouse IgG antibodies (Gene Tex, Irvine, CA, USA). PP2, bisindolylmaleimide I, PD98059, and Bay 11-7082 were purchased from MedChemExpress (Monmouth Junction, NJ, USA). Spectra/Por^®^ dialysis membrane bags (MWCO: 12–14 kDa) were purchased from Spectrum Chemical (New Brunswick, NJ, USA). The listed pharmacological inhibitors did not show any critical cytotoxic effects at the indicated concentrations. All other reagents were of the highest purity commercially available and were used as received.

### 2.2. Culture of Human UCB-MSCs

Human UCB-MSCs were cultured without a feeder layer in the α-minimum essential medium (α-MEM; Thermo, Waltham, MA, USA). The cells were grown in 1% penicillin, 1% streptomycin, and 10% FBS. For each experiment, cells were grown in both the plates and culture dishes with diameters of 35, 60 or 100 mm in an incubator maintained at 37 °C with 5% CO_2_. The medium was replaced with serum-free α-MEM at least 24 h before the experiments. Following incubation, the cells were washed twice with phosphate-buffered saline (PBS) and then maintained in serum-free α-MEM, including all supplements and indicated agents.

### 2.3. Preparation of the Curcumin Nanosphere (CN)

We prepared the curcumin nanospheres (CNs) loaded with curcumin as described previously [16,17]. Briefly, curcumin (5 mg/mL) dissolved in 20 mL of toluene was added dropwise to boiling water (50 mL) under continuous ultrasonication with a frequency of 50 kHz. This mixture was then stirred at 1000× *g* for 20 min, followed by concentration under reduced pressure at 40 °C using a rotary evaporator. The samples were collected and lyophilized to obtain curcumin nanoparticles (CP). A lecithin mixture consisting of lecithin (0.2 mg) and dichloromethane (40 µL) was mixed with the curcumin nanoparticles in a ratio of 1:1 under constant stirring. The mixture was placed in an ultrasonicator for two hours at 20–30 kHz to obtain a clear orange-colored solution, which is designated as curcumin nanosphere (CN). The CN made up of the primary CP was dried with a freeze dryer (Sam Won, Seoul, Korea) and stored at −70 °C.

### 2.4. Field Emission Scanning Electron Microscope (FE-SEM) Measurements

The surface features of the synthesized CN were monitored using the FE-SEM (JSM-6700F, JEOL Ltd., Seoul, Korea). The aqueous dispersion of CN was spread over a silicon wafer and dried under atmospheric air. The samples were placed in carbon stubs and then coated with a gold-palladium layer to a thickness of 200 Å under vacuum conditions. The spectra of CN showed spherical and uniform shapes with a diameter of less than 130 nm.

### 2.5. Determination of the Particle Size and Zeta Potential

The mean particle size diameter, polydispersity index (PDI), and surface charge (zeta potential) of the CN were measured with a Zetasizer Nano ZS analyzer (Malvern Version 7.02, Malvern Instruments Ltd., Malvern, UK). Amounts of approximately 0.1 mg of CN were dissolved separately in 3 mL of a pH 7.4 solution (normal physiological condition) and ultra-sonicated for approximately 30 min at room temperature before the analysis. The supernatant used here was loaded into a disposable cuvette using a syringe with an attached 0.2 µL filter. The analysis was performed at 25 °C with dynamic light scattering (DLS) detected at a 90° angle. The uniformity of the size distribution was determined from the PDI. All the measurements were taken in a double-distilled water in triplicates (*n* = 3), and all data analyses were conducted in automatic mode.

### 2.6. In Vitro Kinetic Release of Curcumin from CN

The experiment for the in vitro kinetic release of the curcumin from the CN was carried out using a Spectra/Por^®^ dialysis membrane bag. The CN dispersion (1 mL) was placed into the dialysis bag (molecular weight cut-off: 12–14 kDa) and immersed into 100 mL of a release medium (0.01 M phosphate buffer, pH 7.4) in a beaker, with shaking at 50 strokes/min while maintaining the temperature at 37 °C using a water bath. At predetermined intervals, 1 mL of the medium was withdrawn and replaced with fresh medium to prevent the degradation of the curcumin released during the experimental period. At the end of the experiment, the samples were analyzed using an ultraviolet-visible spectroscopy (UV-Vis) spectrophotometer (SPARK, Seestrasse, Männedorf, Switzerland) at 430 nm.

### 2.7. Determination of the Encapsulation Efficiency and Loading Capacity

The encapsulation efficiency (%) of curcumin was estimated as the percentage of curcumin encapsulated in the CN by the following equation: Encapsulation efficiency (%) = 100 − (amount of free curcumin/total amount of added curcumin) × 100, where the amount of free curcumin dissolved in a double-distilled water was determined from the supernatant obtained by centrifugation at 15,000× *g* for 20 min at 25 °C. The supernatant was subjected to spectrophotometric analysis at 430 nm with a UV-Vis spectrophotometer (SPARK), and the curcumin concentration was determined using the established standard curve of curcumin. On the other hand, the precipitate encapsulated with curcumin was dissolved in 1 mL of ethanol with mild stirring for 5 min under magnetically stirred conditions to obtain the loading capacity. The loading capacity (%) of the samples was calculated with the following equation: Loading capacity (%) = (mass of encapsulated curcumin)/(total mass of the precipitate encapsulated with curcumin) × 100, where the mass of the encapsulated curcumin dissolved in ethanol was determined by the standard curve of curcumin.

### 2.8. Determination of CN Stability

In order to evaluate the pH effect on the stability of CN, the pH of the CN solutions was adjusted between 2 and 10 by drop wise addition of 1M HCl and NaOH solutions. The effect of sodium chloride (NaCl) on the stability of CN was determined by adding varying volume (50–250 µL) of 0.1 M NaCl to the CN. The effect of temperature on the stability of CN was studied by heating the CN for 24 h at a temperature of 20, 40, 60 and 80 °C in a water bath. At the end of the experiment, the samples were analyzed using ultraviolet-visible spectroscopy (UV-Vis) spectrophotometer (SPARK) at 430 nm.

### 2.9. Wound-Healing Migration Assay

UCB-MSCs were cultured until 90% confluence in six-well cell culture plates. Serum-starved monolayer cells were scraped with pipette tips to create a cell-free region and were then incubated with a serum-free medium supplemented with curcumin and CN for 24 h. During the incubation step, cell migration was observed with an Olympus FluoView™ 300 confocal microscope (Bio Science Korea, Daegu, Korea) equipped with a 100× objective lens (UPLXAPO10X, Olympus, Tokyo, Japan). The cell numbers that migrate to the denude area were directly counted per random microscopic filed and the averages of the sums of the numbers were calculated. Ten random fields per coverslip were counted.

### 2.10. Lamellipodia Formation and Immunofluorescence Analysis

UCB-MSCs were seeded on glass coverslips coated with Matrigel (BD Biosciences, NJ, USA). Serum-starved confluent monolayer cells were scraped with pipette tips to create a wound field and then incubated with a serum-free medium supplemented with CN for 1 h. To detect the formation of lamellipodia, cells were washed twice with cold PBS, fixed in 4% paraformaldehyde for 10 min, permeabilized in 0.2% Triton X-100 for 7 min, and blocked in PBS containing 5% normal goat serum for 30 min at room temperature. Cells were then stained for F-actin with Alexa Fluor 488 phalloidin conjugate (Thermo Fisher Scientific, Hudson, NH, USA) and counterstained with either 4′,6-diamidino-2-phenylindole (DAPI) or propidium iodide (PI) for 30 min to detect the nucleus. After washing with PBS, the samples were mounted on slides with ProLong Gold Antifade Reagent (Invitrogen, Carlsbad, CA, USA) and visualized with an Olympus FluoView™ 300 confocal microscope with a 400× objective lens (UPLXAPO40X, Olympus).

### 2.11. Cellular Uptake of CN

The cellular uptake of the CN and curcumin was studied using fluorescence microscopy with human UCB-MSCs. UCB-MSCs were seeded in a six-well plate at a density of 2 × 10^4^ cells per well and were incubated for 24 h to allow attachment. The medium was removed and the cells were washed with Dulbecco’s phosphate buffered saline (PBS) and treated with the α-MEM medium containing the CN and curcumin at concentrations of 100 pg/mL for 1 h at 37 °C. The cells were rinsed three times with PBS to wash out the CN remaining on the surface of the cells completely. The cells were then stained either for F-actin with Alexa Fluor 555 phalloidin conjugate (Thermo Fisher Scientific) or for curcumin with 2-aminoethyl diphenyl borate (DPBA), which is one of the stabilizing agents for phenolic compounds, used as described previously [27] and counterstained with 4′,6-diamidino-2-phenylindole (DAPI) to detect the nucleus for two hours. After washing with PBS, the samples were mounted on slides with the ProLong Gold Antifade Reagent (Invitrogen) and visualized with an Olympus FluoView™ 300 confocal microscope with a 400 × objective lens (UPLXAPO40X, Olympus).

### 2.12. Transwell Migration Assay

An in vitro transwell migration assay was conducted using a Transwell permeable support with 8.0 µm pore size membrane coated with Matrigel (BD Biosciences) according to the manufacturer’s instruction. Cell suspensions (5 × 10^4^ cells/mL) were placed into the upper chamber in 0.2 mL of serum-free medium. The lower compartment was filled with 0.6 mL serum-free medium containing the CN. After incubation for 24 h, cells that had migrated to the lower surface of the filters were fixed in acetone for 5 min at room temperature and visualized using the H&E staining method.

### 2.13. Western Blot Analysis

Cells were harvested, washed with PBS, and lysed with RIPA buffer (ATTO Corp., Tokyo, Japan) for 30 min on ice. Protein concentrations were determined by BCA protein assay kits (Pierce, Rockford, IL, USA). Equal amounts of protein (20 µg) were resolved by 8–12% sodium dodecyl sulfate polyacrylamide gel electrophoresis (SDS-PAGE) and transferred to polyvinylidene fluoride membranes. The membranes were washed with TBST solution [Tween-20 (0.05%), 10 mM Tris-HCl (pH 7.6), and 150 mM NaCl], blocked with skim milk (5%) for 30 min, and incubated with the appropriate primary antibody at 4 °C overnight. Each membrane was then incubated with a horseradish peroxidase-conjugated secondary antibody for 2 h. The bands were visualized by enhanced chemiluminescence (Amersham Pharmacia Biotech Inc., Piscataway, NJ, USA) and detected by using the Bio-Rad Chemi Doc™ XRS+ System (Bio-Rad, Hercules, CA, USA). The results of the western blot analysis were calculated in terms of relative intensity, using Scion imaging software (Scion Image Beta 4.02, Frederick, MD, USA).

### 2.14. Cell Proliferation Assay

A cell proliferation assay was conducted using the EZ-CYTOX cell proliferation kit (DoGen Bio, Seoul, Korea) according to the manufacturer’s instructions. Cells were cultured on 96-well culture plates. After incubation with CN, 10 µL of the EZ-CYTOX master mix was added to each well and left for 1 h. Cell proliferation was analyzed by measuring the absorbance at 450 nm.

### 2.15. Mouse Skin Wound Healing Model

All experimental procedures were approved by Daegu Hanny University, and all animal procedures were performed following the National Institutes of Health Guidelines for the Humane Treatment of Animals, with approval from the Institutional Animal Care and Use Committee of Daegu Hanny University (DHU-2020-001) at 10 February 2020. Eight-week-old male ICR mice were used. All surgeries were performed under anesthesia using a 2:1 mixture of Zoletil™ (20 mg/kg, Virbac Laboratories, Carros, France) and Xylazine HCl (10 mg/kg, Rompun^®^, Bayer, Germany) and all efforts were made to minimize suffering. Mouse skin wounding and stem-cell implantation were performed as described previously [28,29]. Briefly, after shaving the backs and scrubbing with an organic iodine solution, a circular full-thickness wound was surgically created using a 6-mm-diameter sterile biopsy punch. UCB-MSCs were pre-treated with 100 pg/mL CN for 24 h prior to skin transplantation. To determine the functional role of UCB-MSCs pre-treated with CN, experimental animals were divided into five groups: Vehicle groups that received a PBS alone (group 1, *n* = 8), UCB-MSCs transplantation groups that were given UCB-MSCs treated either with PBS as a control (group 2, *n* = 8) or with 100 pg/mL of CN (group 3, *n* = 8); and UCB-MSCs/c-Src inhibitor (PP2) transplantation groups that were given UCB-MSCs pretreated with a Src inhibitor, PP2 (10 µM), prior to exposure to 100 pg/mL CN (group 4, *n* = 8) or a PBS (group 5, *n* = 8) for 24 h. We injected 1 × 10^6^ UCB-MSCs in 70 µL PBS containing 50% growth-factor–reduced Matrigel (BD Biosciences, NJ, USA) into the dermis at two sites around the wound and topically applied 0.3 × 10^6^ UCB-MSCs in 30 µL PBS containing 50% Matrigel onto the wound bed on day 0 and day 8. Subsequently, the wounds were dressed with Tegaderm (3M, London, ON, Canada). Images of the wounds were taken on days 0, 8 and 12 with a digital camera system (D50, Nikon, Tokyo, Japan) at the same camera/subject distance (30 cm). The wound closure sizes were determined by wound resealing measurements from the images captured at the wounded sites. The wound areas were measured planimetrically using the free-hand tool in the Image J software (NIH, Bethesda, MD, USA). Percent wound closure values were calculated as the difference in the wound size on a particular day compared to day 0 (time of wounding)/initial wound size. On day 12, the wound tissues were embedded in O.C.T. compound (Sakura Finetek, Torrance, CA, USA) and stored at −70 °C, after which the samples were cut into 6-µm-thick frozen sections using a cryosectioning machine and mounted on SuperFrost Plus slides (Thermo Fisher Scientific, Rockford, IL, USA) for hematoxylin and eosin (H&E) staining. The quantitative aspects of the score were evaluated according to the percentage of the tissue presenting the specific qualitative features of re-epithelialization during the process of skin wound healing according to a method published by Rajabi et al. [30] (Table 1) and validated previously through similar experimental models [31,32], after which the averages of the sums of the scores for each mouse were calculated. A histological analysis was carried out by four independent observers who were unaware of the treatment. On the other hand, we have further checked the endotoxin contamination in CN and UCB-MSCs treated with CN by using endotoxin quantitation kit (Pierce™ LAL Chromogenic Endotoxin Quantitation Kit, Thermo). The level of endotoxin was 0.014 ± 0.005 and 0.018 ± 0.001 EU/mL at 100 pg/mL of CN and 10 µg/mL of UCB-MSCs treated with 100 pg/mL of CN, respectively. This means that CN and UCB-MSCs primed by CN are safety and purified products containing very low level of endotoxin (less than 0.02 EU/mL) and suitable for our experiments in this study.

### 2.16. Statistical Analysis

Results are expressed as means ± standard errors (S.E.). All experiments were analyzed by one-way analysis of variance (ANOVA), followed in some cases by a comparison of treatment means with a control using the Bonferroni-Dunn test. Differences were considered statistically significant at *p* < 0.05.

## 3. Results

### 3.1. Characterization of the Curcumin Nanosphere (CN)

**Figure 1:**
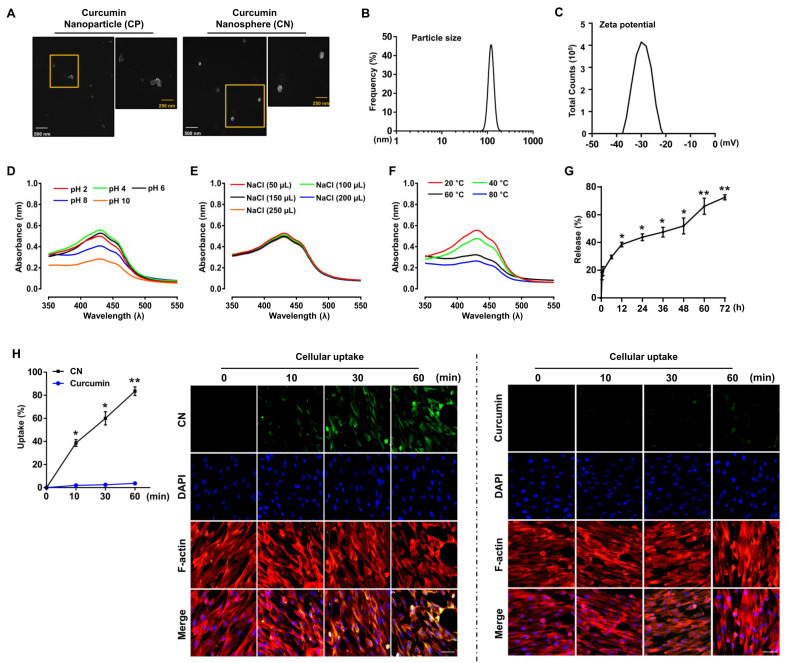
Characterization of curcumin nanosphere (CN). (**A**) Field emission scanning electron microscope (FE-SEM) analysis of curcumin nanoparticle (CP) and CN. The white and yellow scale bars indicate 500 nm and 250 nm, respectively *n* = 3. (**B**) Particle size analysis of CN by using dynamic light scattering method. *n* = 3. (**C**) Zeta potential analysis of CN. *n* = 3. (**D**) UV-Vis absorption spectra showing the effect of varying pH (2, 4, 6, 8 and 10) on the stability of CN. n = 3. (**E**) UV-Vis absorption spectra showing the effect of different volume (50–250 µL) of 0.1 M NaCl on the stability of CN. *n* = 3. (**F**) UV-Vis spectra showing the effect of temperature (20, 40, 60 and 80 °C) on the stability of CN. *n* = 3. (**G**) In vitro kinetic release of curcumin from CN in PBS (pH 7.4) at 37 °C. *n* = 3. Data represent means ± S.E. *n* = 3. * *p* ≤ 0.05 versus 0 h. ** *p* ≤ 0.01 versus 0 h. (**H**) The cellular uptake of the CN and curcumin for 1 h was quantified (left panel) and visualized by staining the CN and curcumin with 2-aminoethyl diphenyl borate (DPBA) (right panel). Data represent means ± S.E. *n* = 3. * *p* ≤ 0.01 versus 0 min. ** *p* ≤ 0.05 versus 0 min. Green fluorescence represents the cellular uptake of CN and curcumin labeled with DPBA. 4′,6-diamidino-2-phenylindole (DAPI) was used for nuclear counterstaining (Blue). Scale bars represent 100 μm (magnification, × 400).

We previously identified that the curcumin nanosphere (CN) displays a sharp absorption peak around a wavelength of 430 nm and all the signature functional groups known to be characteristic of curcumin, and that it has a water-solubility 160 times greater than that of curcumin [16]. In the present study, we also confirmed that the polygonal and irregular shapes of the primary curcumin nanoparticle (CP) surface as monitored by field emission scanning electron microscopy (FE-SEM) can be transformed into spherical and uniform nanostructures (CN) by using a lecithin-based nano-emulsification method (Figure 1A). To characterize the CN further, dynamic light scattering (DLS) and zeta potential measurements were used to ascertain the particle size, surface charge, and homogeneity in the particle population. We found that the average particle size, zeta potential, and polydispersity index (PDI) of the CN were 127.3 ± 14.8 nm (Figure 1B), −29.3 ± 5.7 mV (Figure 1C) and 0.35 ± 0.1 (data not shown), respectively. We further determined the encapsulation efficiency and loading capacity of the CN (Table 2). We found that the encapsulation efficiency of the CN was 87.1 ± 0.1% and the loading capacity was 9.0 ± 0.1%. These findings mean that curcumin nanospheres are a very homogeneous nanoparticulate suspension with diameters around 120 nm that are successfully entrapped into the lecithin and that they are highly stable with a negative charge when delivered to the target cells.

We also evaluated the stability of CN while varying the pH and ionic strengths and within a range of temperatures. As shown in Figure 1D, CN was stable at extremely acidic pHs, i.e., 2–4, and showed enhanced stability at pH values in the range of 4–6. Moderate stability of the CN was observed at pH 8, where the CN showed a less significant red shift and peak broadening. However, decreased stability was observed at pH 10. These results suggest that CN is stable over a wide range of pH, although there was some aggregation of the CN, leading to decreased absorption at an extremely alkaline pH. The stability of CN against different ionic strengths was determined using different volumes of NaCl (Figure 1E). There was only a slight effect on the CN when changing the volume of 0.1 M NaCl from 50 μL to 250 μL, indicating that the resulting nanospheres are stable at these conditions. The effect of the temperature on the stability of CN was also investigated by heating the nanoparticle solution for 24 h from 20 to 80 °C (Figure 1F). A slight decrease in the absorbance at 60-80 °C was observed, indicative of the temperature-facilitated aggregation of CN. However, the CN still showed the typical absorption peak at 430 nm, suggesting that it has remarkable thermal stability at elevated temperatures. In addition, we attempted to determine the drug release kinetics of CN during a time of 72 h (Figure 1G). We found that 39.5 ± 0.93% of curcumin was released from the CN in the first 12 h. After 48 h, the amount of curcumin released was 51.9 ± 3.31%, and this level finally reached 72.6 ± 1.04% in 72 h, demonstrating a long-lasting release pattern. The cellular uptake of CN by UCB-MSCs was confirmed by confocal microscopy. As show in Figure 1H, the cellular uptake of CN by UCB-MSCs was >83.5 ± 2.18% at 60 min, which is about 23 times higher than that of curcumin.

### 3.2. The Effect of CN on Migration of Human UCB-MSCs

**Figure 2:**
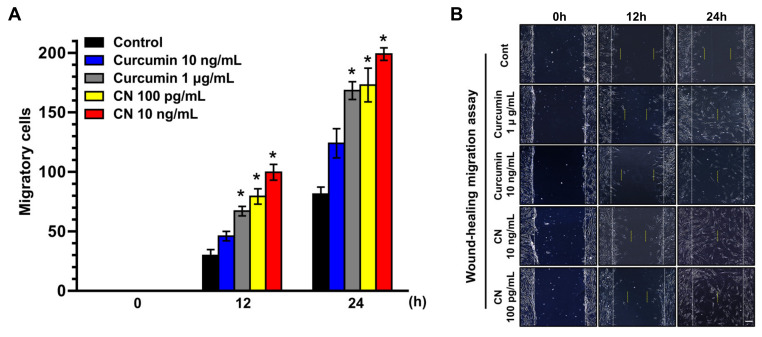

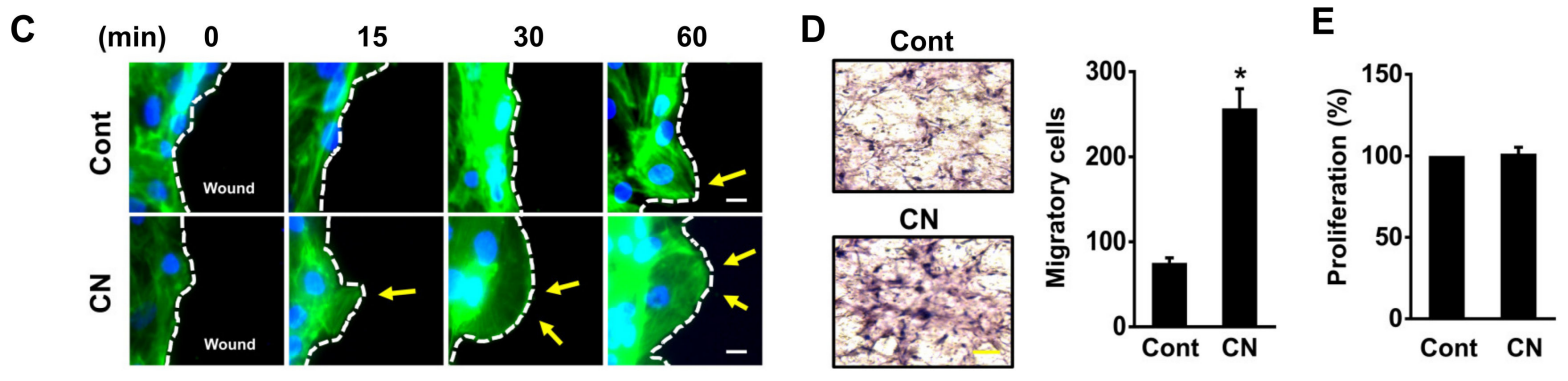
The effect of CN on migration of human UCB-MSCs. (**A**,**B**) Dose and time responses of Curcumin (10 ng/mL and 1 µg/mL) and CN (100 pg/mL and 10 ng/mL) in the migration of UCB-MSCs were quantified and visualized with the confocal microscopy. Scale bar represents 100 μm (magnification, × 100). * *p* ≤ 0.01 versus control. (**C**) Lamellipodial extrusions in cells treated with CN of 100 pg/mL for 60 min are shown. Cells were fixed and labeled with phalloidin-Alexa Fluor 488 (green) to identify the leading edge of lamellipodia (arrows). Dashed lines indicate the leading edges. *n* = 3. Scale bars represent 100 μm (magnification, × 400). (**D**) The migration capacity of UCB-MSCs treated with CN for 24 h was assessed in transwell permeable support with 8.0 μm pore size membrane coated with Matrigel. Data represent the means ± S.E. *n* = 3. Scale bars represent 100 μm (magnification, × 200). * *p* ≤ 0.001 versus control. (**E**) The proliferation of UCB-MSCs treated with CN for 24 h was determined by EZ-CYTOX cell proliferation kit. Data represent the means ± S.E. *n* = 3.

The clinical application of curcumin has been restricted due to its poor aqueous solubility, permeability and bioavailability. To evaluate the role of CN loaded with curcumin in the regulation of stem cell motility, UCB-MSCs were exposed to various concentrations of CN and curcumin for 24 h. A significant increase in the cell migration was observed starting at 12 h after incubation with 100 pg/mL of CN (Figure 2A). On the other hand, the migration-promoting effect of curcumin was observed starting at an amount of 1 µg/mL. These results indicate that CN has greater promotive efficacy by 10,000-fold on the cell motility than curcumin. The same results were visually confirmed with a microscope (Figure 2B).

Lamellipodial protrusions play an important role in forwarding cell movement [33]. Prominent F-actin-rich lamellipodial protrusions were observed following 15 min of CN treatment (Figure 2C). In addition, we found that CN significantly increases the number of cells that are migrated to the lower surface of the membrane coated with Matrigel (Figure 2D), but it did not affect the cell proliferation (Figure 2E). These results suggest that the effect of CN in the stem cell motility is an independent process of the cell proliferation.

### 3.3. CN Regulates the Activation of c-Src and PKC

**Figure 3:**
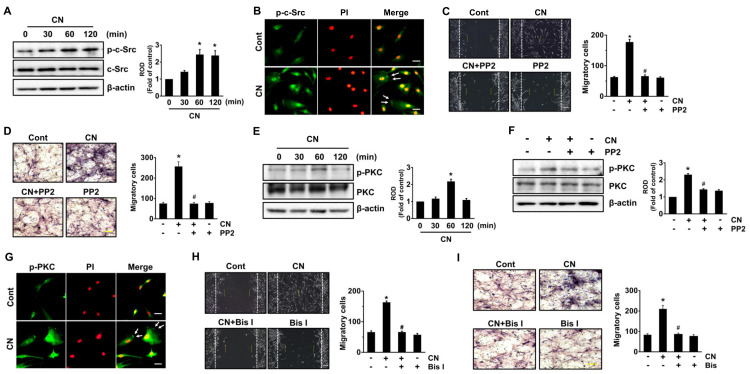
CN regulates the activation of c-Src and PKC. (**A**) Time responses of phosphorylation of c-Src in cells treated with CN are shown. Data represent the means ± S.E. *n* = 3. * *p* ≤ 0.001 vs. 0 min. (**B**) Membrane translocation of p-c-Src (green) was determined by confocal microscopy. Propidium iodide (PI) was used for nuclear counterstaining (red). *n* = 3. Scale bars represent 100 μm (magnification, × 400). (**C**) Cells were pretreated with c-Src inhibitor, PP2 (10 µM) for 30 min prior exposure to CN for 24 h. Cell migration was determined by wound-healing migration assay (left panel) and quantified (right panel). Scale bar represents 100 µm (magnification, × 100). *n* = 3. * *p* ≤ 0.001 vs. control. # *p* ≤ 0.05 vs. CN alone. (**D**) The migration capacity of UCB-MSCs treated with CN for 24 h was assessed in transwell permeable support with 8.0 μm pore size membrane coated with Matrigel. Data represent the means ± S.E. *n* = 3. Scale bars represent 100 μm (magnification, × 200). * *p* ≤ 0.01 vs. control. # *p* ≤ 0.01 vs. CN alone. (**E**) Time responses of phosphorylation of p-PKC in cells treated with CN are shown. Data represent the means ± S.E. *n* = 3. * *p* ≤ 0.001 vs. control. (**F**) Cells were pretreated with PP2 for 30 min prior exposure to CN for 60 min. The level of phosphorylation of PKC was determined by western blot. Data represent the means ± S.E. *n* = 3. * *p* ≤ 0.05 vs. control. # *p* ≤ 0.01 vs. CN alone. (**G**) Membrane translocation of p-PKC (green) was determined by confocal microscopy. Propidium iodide (PI) was used for nuclear counterstaining (red). *n* = 3. Scale bars represent 100 μm (magnification, × 400). (**H**) Cells were pretreated with PKC inhibitor, Bisindolylmaleimide I (Bis I, 10 µM) for 30 min prior exposure to CN for 24 h. Cell migration was determined by wound-healing migration assay (left panel) and quantified (right panel). Scale bar represents 100 µm (magnification, × 100). *n* = 3. * *p* ≤ 0.001 vs. control. # *p* ≤ 0.01 vs. CN alone. (**I**) The migration capacity of UCB-MSCs treated with CN for 24 h was assessed in transwell permeable support with 8.0 μm pore size membrane coated with Matrigel. Data represent the means ± S.E. *n* = 3. Scale bars represent 100 μm (magnification, × 200). * *p* ≤ 0.01 vs. control. # *p* ≤ 0.05 vs. CN alone. (**A**,**E**,**F**) ROD is the abbreviation for relative optical density.

To know how CN regulates the stem cell motility, we next evaluated the activation of c-Src tyrosine kinase and protein kinase C (PKC), which are responsible for the cell movement [34,35]. CN (100 pg/mL) significantly induced the phosphorylation of c-Src between 60 and 120 min (Figure 3A). The increased membrane accumulation of p-c-Src in the cell treated with CN was further confirmed by immunofluorescence staining and counter-labeling with propidium iodide (PI) (Figure 3B). Interestingly, a pre-treatment with the c-Src inhibitor, PP2, inhibited the cell migration as induced by CN (Figure 3C). The effect of CN via c-Src in promoting the cell motility was further confirmed by transwell migration assay (Figure 3D). CN significantly induced the phosphorylation of PKC (Figure 3E), though the increase at 60 min could be blocked by c-Src inhibitor (Figure 3F). In addition, translocation of p-PKC from the cytosol to the membrane compartment was observed after cells were treated with CN at 60 min as assessed by immunofluorescence staining (Figure 3G). Importantly, cell migration was abrogated by blockage of PKC with PKC inhibitor, Bisindolylmaleimide I (Bis I), in the cells treated with CN (Figure 3H). The migration-promoting effect of CN via PKC was further assessed by transwell migration assay (Figure 3I). These results indicate that CN regulates the stem cell motility by activation of c-Src and PKC.

### 3.4. CN Uniquely Regulates the ERK Pathway Responsible for the Cell Migration

**Figure 4:**
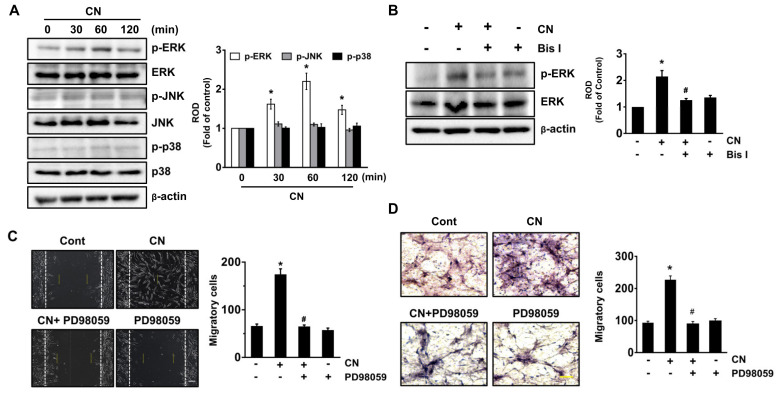
CN uniquely regulates the ERK pathway responsible for the cell migration. (**A**) Time responses of phosphorylation of mitogen-activated protein kinases (MAPK) in cells exposed to CN for 120 min are shown. Data represent the means ± S.E. *n* = 3. * *p* ≤ 0.01 vs. 0 min. (**B**) Cells were pretreated with Bis I for 30 min prior exposure to CN for 60 min. The level of phosphorylation of MAPK was determined by western blot. Data represent the means ± S.E. *n* = 4. * *p* ≤ 0.01 vs. control. # *p* ≤ 0.05 vs. CN alone. (**C**) Cells were pretreated with ERK inhibitor, PD98059 (10 µM) for 30 min prior exposure to CN for 24 h. Cell migration was determined by wound-healing migration assay (left panel) and quantified (right panel). Scale bar represents 100 µm (magnification, × 100). *n* = 3. * *p* ≤ 0.01 vs. control. # *p* ≤ 0.001 vs. CN alone. (**D**) The migration capacity of UCB-MSCs treated with CN for 24 h was assessed in transwell permeable support with 8.0 μm pore size membrane coated with Matrigel. Data represent the means ± S.E. *n* = 3. Scale bars represent 100 μm (magnification, × 200). * *p* ≤ 0.001 vs. control. # *p* ≤ 0.01 vs. CN alone. (**A**,**B**) ROD is the abbreviation for relative optical density.

Mitogen-activated protein kinases (MAPKs) are well-known mediators of cell migration at downstream of PKC. CN significantly increased the phosphorylation of extracellular signal-regulated kinase (ERK) between 30 and 60 min, but it did not affect the activation of either JNK or p38 MAPK (Figure 4A). However, the unique activation of ERK at 60 min was significantly inhibited by a treatment with PKC inhibitor (Figure 4B). Furthermore, pretreatment with the ERK inhibitor, PD98059 significantly blocked the cell migration induced by CN in the models of scratch wound healing (Figure 4C) and transwell migration (Figure 4D). Taken together, the above results suggest that CN in acting on the PKC uniquely stimulates the ERK activation to promote the stem cell motility.

### 3.5. Regulatory Effect of CN on the Activation of NF-κB and the Cytoskeletal Reorganization

**Figure 5:**
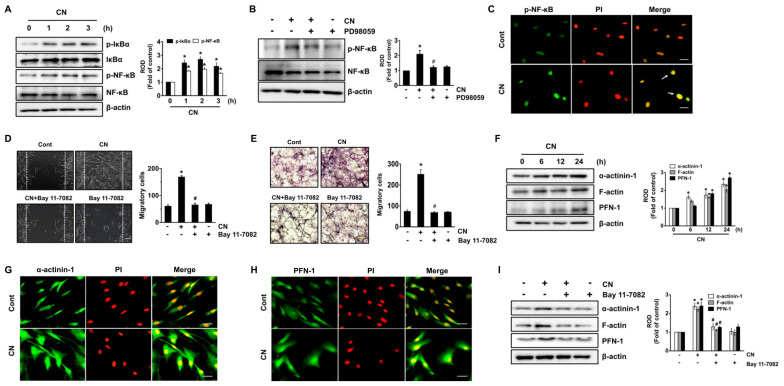
Regulatory effect of CN on the activation of NF-κB and the cytoskeletal reorganization. (**A**) Time responses of phosphorylation of IκBα and NF-κB in cell treated with CN are shown. Phosphorylation of IκBα and NF-κB were determined by western blot. Data represent means ± S.E. *n* = 3. * *p* ≤ 0.01 vs. 0 h. (**B**) Cells were pretreated with PD98059 for 30 min prior exposure to CN for 2 h. The level of phosphorylation of NF-κB was determined by western blot. Data represent the means ± S.E. *n* = 4. * *p* ≤ 0.01 vs. control. # *p* ≤ 0.05 vs. CN alone. (**C**) The increased level of p-NF-κB (green) was determined by confocal microscopy. Propidium iodide (PI) was used for nuclear counterstaining (red). *n* = 3. Scale bars represent 100 μm (magnification, × 400). (**D**) Cells were pretreated with NF-κB inhibitor, Bay 11-7082 (10 µM) for 30 min prior exposure to CN for 24 h. Cell migration was determined by wound-healing migration assay (left panel) and quantified (right panel). Scale bar represents 100 µm (magnification, × 100). *n* = 3. * *p* ≤ 0.01 vs. control. # *p* ≤ 0.05 vs. CN alone. (**E**) The migration capacity of UCB-MSCs treated with CN for 24 h was assessed in transwell permeable support with 8.0-μm pore size membrane coated with Matrigel. Data represent the means ± S.E. *n* = 3. Scale bars represent 100 μm (magnification, × 200). * *p* ≤ 0.01 vs. control. # *p* ≤ 0.05 vs. CN alone. (**F**) Time responses of CN in the expression of α-actinin, PFN-1, and F-actin are shown (0–24 h). Data represent means ± S.E. *n* = 3. * *p* ≤ 0.01 vs. 0 h. The expression of α-actinin (green) (**G**) and PFN-1(green) (**H**) was determined by confocal microscopy. PI was used for nuclear counterstaining (red). *n* = 3. Scale bars represent 100 μm (magnification, × 400). (**I**) Cells were pretreated with Bay 11-7082 for 30 min prior exposure to CN for 24 h. Expression of α-actinin, F-actin, and PFN-1 were determined by western blot. Data represent the means ± S.E. *n* = 4. * *p* ≤ 0.01 vs. control. # *p* ≤ 0.05 vs. CN alone. (**A**,**B**,**F**,**I**) ROD is the abbreviation for relative optical density.

We further examined the role of CN in activation of nuclear factor-kappa B (NF-κB), an important transcription factor related to the genes expression of cytoskeletal reorganization [36]. CN significantly induced the phosphorylation of NF-κB inhibitor (IκBα) and NF-κB from 1 to 3 h (Figure 5A), though the increase at 2 h could be blocked by ERK inhibitor (Figure 5B). The increased level of p-NF-κB induced by CN was also confirmed by the immunofluorescence method (Figure 5C). Interestingly, the cell motility triggered by CN was significantly inhibited by a treatment with the NF-κB inhibitor, Bay 11-7082 (Figure 5D,E). These results demonstrate the ERK-mediated NF-κB activation is a critical step to regulate the cell migration induced by CN. We then determined whether CN regulates the expression of cytoskeletal reorganization-related proteins. The levels of α-actinin-1, filamentous (F)-actin, and profilin (PFN)-1, which are believed to be essential in the dynamic regulation of actin filament remodeling at cell surfaces were significantly augmented by 2.34, 2.03 and 2.71-fold at 24 h after treatment with 100 pg/mL of CN, compared to the control, respectively (Figure 5F). The expression of α-actinin-1 and PFN-1 was further confirmed by immunofluorescence staining in CN-treated UCB-MSCs (Figure 5G,H). To verify the role of signaling mediators in the promoting cell motility, we have considered whether the inhibition of NF-κB influences on the cell migration of UCB-MSCs for 24 h. Interestingly, a pretreatment with the NF-κB inhibitor significantly blocked the expression of cytoskeletal reorganization-related proteins induced by CN (Figure 5I). These results indicate that CN induces the NF-κB-mediated expression of α-actinin, F-actin, and PFN-1 during the promotion of cell migration.

### 3.6. The Role of CN in Mouse Model for Skin Wound Healing

**Figure 6:**
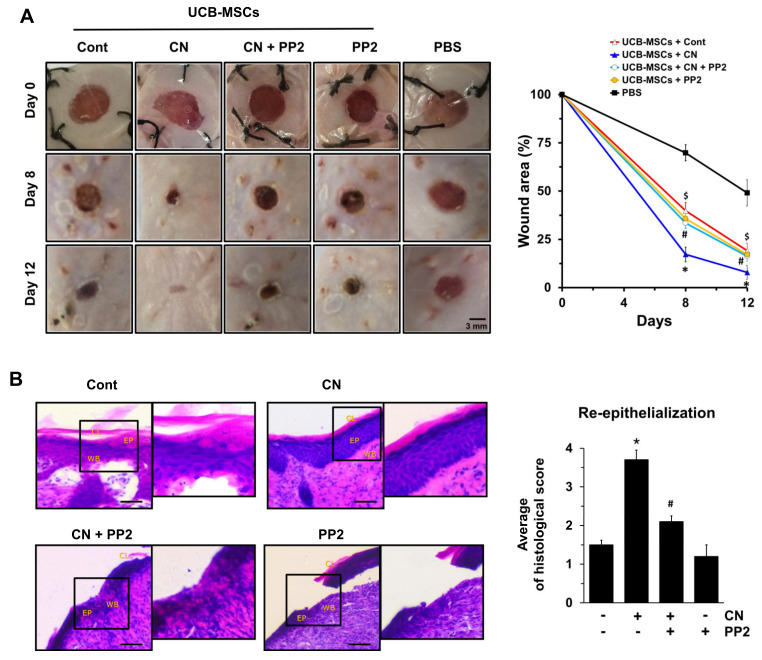
The role of CN in mouse model for skin wound healing. (**A**) Representative gross images on skin wound healing on day 0, 8, and 12 are shown (left panel). Mouse skin wounds were made by 6-mm-diameter biopsy punch. For the stem-cell implantation, mice were given PBS alone or UCB-MSCs treated with Cont, CN, CN+PP2, and PP2. Quantifications of wound sizes relative to original wound size for 12 days are shown. Data represent the mean ± S.E. *n* = 8. ^$^, *p* < 0.01 vs. PBS alone. * *p* ≤ 0.01 vs. cont. # *p* ≤ 0.05 vs. CN alone. (**B**) Representative wound tissues stained with H&E on day 12 are shown (left panel). Scale bars, 100 μm. Abbreviations: EP, epidermis; WB, wound bed; CL, cornified layer. Histological scores in re-epithelialization (right panel) were quantified according to the Table 1. Data represent the mean ± S.E. *n* = 8. * *p* ≤ 0.001 vs. cont. # *p* ≤ 0.01 vs. CN alone.

To ensure the functional roles of CN in promoting of the stem cell motility, we have further investigated the effect of human UCB-MSCs treated with CN on skin wound healing in mice. There were clear wound healings in mice treated with human UCB-MSCs alone for 12 days and the percentages of the wound area were significantly decreased, compared to the PBS alone. On day 12, however, the cutaneous wound repair was significantly accelerated in the mice group that received UCB-MSCs treated with CN, compared with the control (Figure 6A). Interestingly, the mice group that received UCB-MSCs treated with c-Src inhibitor, PP2, showed a significant delay in wound healing despite the CN pre-treatment. This result indicates the functional role of c-Src in promoting cell motility orchestrated by CN in the mouse skin wound healing model. A histologic examination on day 12 showed that the transplantation of UCB-MSCs, pre-treated with CN, led to the nearly complete restoration of re-epithelialization from a mechanical skin wound (Figure 6B). However, the wound bed was still not completely covered with epidermis and a cornified layer in mice treated with UCB-MSCs pretreated with PP2 prior to the exposure to CN.

## 4. Discussion

In the present study we show that nanospheres loaded with curcumin (CN) trigger the phosphorylation of c-Src to regulate PKC activation, where CN uniquely regulates the phosphorylation ERK to facilitate NF-κB-mediated stem cell motility, which plays a critical role in the promotion of the mouse skin wound healing process. Despite the wealth of evidence indicating that curcumin supplements can be considered safe when taken at recommended doses and that they elicit diverse biological/pharmacological effects, the broader clinical application of curcumin has remained limited due to its hydrophobicity and low bioavailability [37]. To enhance its bioavailability, curcumin has been encapsulated with different formulations using hydrogels, micelles, and nanoparticles [37]. With regard to nano-formulations of curcumin, it has been reported that curcumin nanoparticles (CP) made up primarily of curcumin show improved more than three-fold better bioavailability relative to curcumin alone [38]. Moreover, it was proven that controlling the shape of CP with a lipid nano-carrier is a promising strategy for the realization of a feasible drug-delivery system that can stabilize the physical and chemical properties of curcumin in terms of cellular uptake, biodistribution, and accumulation at sites of interest [39]. In agreement with the above reports, our data indicate that nanospheres loaded with curcumin (CN) with diameters of less than 120 nm have good nanoparticle size distribution, encapsulation efficiency, and loading capacity, and possess the remarkable stability over a wide range of pH, ionic strength, and temperature. In addition, CN exhibited a negative charge due to the use of a lipid nanocarrier (lecithin) with high stability, responsible for attachment onto the cell plasma membrane, cellular uptake, and bioavailability of the anionic CN. These results are further supported by previous evidences showing that lower PDI values are related to more monodisperse nanoparticles and greater particle stability, while zeta-potential values higher than +20 and lower than −20 mV are normally associated with high stability of nanoparticles from particle aggregations [40,41]. Concerning the structure of CN, we previously have shown the chemical structures of the CN by using the FT-IR spectroscopy and reported that there are many interactions of the phenolic-OH of curcumin with the lecithin [16]. These results indicate that CN is a nanostructure made of amphiphilic lecithin that gathers by itself to form a core/shell structure in the aqueous solution. This means that the hydrophobic core of CN is loaded with hydrophobic curcumin nanoparticles, at the same time the hydrophilic shell makes the whole system soluble in water and stabilizes the core. Since CN is a lecithin liposome-based drug delivery system composed of a hydrophobic core loaded with curcumin nanoparticle and a hydrophilic shell, the mechanism of the in vitro cellular uptake of the CN can be assumed to be the spontaneous membrane fusion and endocytosis [42]. Thus, we have considered the short-term time scales less than 1 h as an appropriate starting point for the endocytic uptake of CN that stimulates the phosphorylation cascade of intracellular proteins. Due to the existence of lipids on the surface of curcumin nanoparticles, which are similar to the cell membrane component, the uptake of the CN is facilitated by the mutual interaction between the CN and the cell membrane. Many researchers have reported that liposomes are taken up into cells via clathrin-mediated endocytosis [43,44]. In addition, it has been also suggested that around 100 nm-sized nanoparticles mainly are internalized via clathrin-mediated endocytosis [45]. Thus, we speculate that spherical CN with diameters around 120 nm is preferably suspected to internalize through a clathrin-dependent pathway. Following entry into the cell, the internalized CN might be transported through early endosomes to the sorting endosomes. A fraction of CN recycles back to the cell exterior while another CN is transported to secondary endosomes/lysosomes from where curcumin nanoparticles escape into the cytoplasm [46,47]. Curcumin nanoparticles that escape into the cytoplasm should act as intracellular reservoirs for sustained release of the encapsulated therapeutic agent. In agreement with the above mechanisms, our data indicate that the release of curcumin from CN occurs in a sustained manner over 72 h, suggesting that CN is a sustained drug delivery system with a prolonged therapeutic effect on cytoskeletal reorganization process to promote stem cell motility.

We subsequently showed that CN acts as a strong stimulator of UCB-MSC motility accompanied by lamellipodial protrusion, which plays an important role in forward cell movement. Moreover, we found that the migration-promoting efficacy of CN was 10,000-fold higher than that of naive curcumin, suggesting that CN can function as an effective drug-delivery system to improve the bioavailability of curcumin in the UCB-MSCs while also having pharmacological functions by promoting cytoskeletal reorganization during the wound-healing process. Given that the plasma membrane is directly linked to and functionally integrated with the underlying actin based cytoskeleton, it may be anticipated that endocytosis of CN would also require the disruption of the cell membrane, and the rearrangement of the actin by a process called micropinocytosis. In agreement with this, our results in the present study showed that CN significantly increases F-actin-rich lamellipodial protrusions and cytoskeleton reorganization responsible for the cell migration. Although micron size particles are generally known to internalize the cells via macropinocytosis [48], most of the literature reports highlight that the nanoparticles undergo cellular internalization via more than one endocytic pathway. Recently, Zhang et al. [49] reported that lapatinib loaded nanoparticles with diameters of less than 60 nm are internalized into BT-474 cells through energy-dependent endocytosis involving clathrin-dependent pinocytosis and macropinocytosis. In addition to the clathrin-mediated endocytosis, therefore, we suggest that CN could activate a micropinocytotic internalization and leads to an increment of the actin polymerization, actin-mediated ruffling, and macropinosome formation, which are required for the cell migration and the wound healing process. Concerning the cellular mechanisms of CN with regard to amplifying stem cell bioactivity, we showed a unique relationship between the c-Src signaling pathway and PKC activation which are responsible for the phosphorylation of extracellular signal-regulated kinases (ERK) in the regulation of UCB-MSC migration. We have previously have reported that c-Src, PKC, and ERK are key immediate downstream effectors of Inα6β4 and that their activities within 2 h are required for Inα6β4 signaling competency in promoting of the stem migration and proliferation [50,51]. Upon phosphorylation, we thought that the key effectors would distribute the signal downstream to over a hundred substrates responsible for the cell migration for 12~24 h. These results are also consistent with the previous our results showing that melatonin and netrin-1 significantly induce the cell migration of UCB-MSCs within 24 h by stimulating the cytoskeletal reorganization process where the key effectors link to other molecules that are important in the cell migration process [50]. c-Src, a non-receptor tyrosine kinase, acts as an effector of integrin and/or tyrosine kinase receptor signaling, where it plays a key role in controlling membrane protrusion during cell movement [52]. c-Src, together with focal adhesion kinase (FAK), has been clearly shown to govern the formation of stress fibers and focal adhesions of discrete adhesive plaques containing numerous structural (e.g., α-actinin and vinculin) and signaling molecules (e.g., FAK, RhoA, integrins and paxillin) [53]. These results provide strong evidence that CN coupled with c-Src is an important microenvironmental cue in the triggering of UCB-MSC motility. We also observed that CN induces PKC translocation and phosphorylation, in that PKC is required for c-Src activation to promote UCB-MSC motility. Indeed, the Src family has been shown to phosphorylate PKC on specific tyrosine residues [54]. PKC, a serine/threonine kinase, is enriched during focal adhesion, where it plays a key role in the connection with the actin cytoskeleton by interacting with many prominent substrates, such as α-actinin, Rho GTPases, vinculin, and talin [34]. Although PKC is an important kinase responsible for the growth and survival of stem cells [55], many studies have also established a critical role of PKC in directional migration [56,57] as well as in hematopoietic stem cell motility [58]. Having shown that c-Src and PKC are major effector molecules of CN signaling pathways in UCB-MSCs, we also attempted to uncover the mechanism by which these effectors link to other molecules that are important in the cell migration process. We found that CN increases ERK phosphorylation via the activation of PKC. This means that PKC with c-Src acts to transduce CN signals to ERK cascades. However, JNK and p38 MAPK are not activated by a CN treatment, implying that ERK is a relevant target linked to cytoskeletal reorganization as mediated by PKC during the wound-healing process. Thus, these results provide convincing proof that the coordination of the c-Src signaling pathway and PKC activation is required for human UCB-MSC motility induced by CN through the facilitation of the distinct activation of ERK pathways.

In relation to how ERK stimulates stem cell migration, our study revealed that CN facilitates the phosphorylation of nuclear factor-κB (NF-κB) and that the blocking of NF-κB reduces the wound healing capacity of CN, suggesting that NF-κB activation is involved in this process. NF-κB is an ubiquitous transcription factor that translocates from the cytoplasm into the nucleus upon the phosphorylation/degradation of the inhibitor κBα (IκBα); it has also been shown to regulate multiple physiological processes, including stem cell migration and differentiation [59,60]. Regarding the role of ERK in NF-κB activation, earlier work showed that pERK1/2 has the ability to translocate into the nucleus, where it phosphorylates various substrates, such as transcriptional factors, thereby transmitting the signals received by cell surface receptors to the nucleus [61]. Hence, it is conceivable that CN has a potential role in promoting the NF-κB pathway through the activation of ERK. These findings suggest that NF-κB is an important transcriptional regulator of the ERK signaling pathway to regulate stem cell migration as induced by CN. In support of these findings, our results revealed that CN activates NF-κB to elevate the levels of α-actinin, profilin (PFN)-1, and filamentous (F)-actin, which are known to play important roles in cytoskeletal reorganization and lamellipodial protrusion during the wound-healing process [62,63,64]. It was clearly shown that PFN-1 is active during the enhancement of the actin assembly at the plasma membrane, thereby increasing F-actin expression, which drives cell motility and other actin-linked processes [62,63]. Specifically, α-actinin cross-links F-actin into bundles to form a cytoskeletal network [64]. Hence, our results are consistent with the notion that CN enhances the transcriptional targeting of NF-κB for close cooperation with the cell migration machinery to amplify stem cell bioactivity.

Finally, we showed convincing in vivo proof that the transplantation of UCB-MSCs pre-treated with CN improves skin wound healing, where it has the ability to enhance epidermal reorganization and restore the normal tissue microarchitecture by regulating stem cell migration. Thus, UCB-MSCs may be more useful if they are pre-activated by CN, as doing so can demonstrate the potential benefits of stem cell pre-activation not only with timely efficacy, but also with a reduction of the side effects of an overdose of CN. The pre-activation of UCB-MSCs may offer a means of improving the potency of these cells without the need for additional cell numbers. Concerning the fate of UCB-MSCs, we previously have shown that UCB-MSCs labeled with 5-bromo-2’-deoxyuridine (BrdU) migrate to the wound sites in a mouse skin excisional wound model [65]. In addition, the majority of mesenchymal stem cells have been shown to enhance the regeneration of damaged tissues via a paracrine mechanism rather than through multi-lineage differentiation [3,66]. Thus, it is possible that the transplantation of UCB-MSCs pre-treated with CN migrate into the wound sites where it enhances paracrine mechanisms to promote the wound closure, granulation, and re-epithelialization of injured tissues. These results are consistent with the notion that MSCs treated with TNF-α stimulate the tissue regeneration process through paracrine mechanisms [66]. Moreover, we showed that the inhibition of c-Src in UCB-MSCs pre-activated with CN failed to regulate the repair of skin wounds. Therefore, our findings elucidate novel roles of c-Src induced by CN with regard to UCB-MSC motility to enhance the re-epithelialization of injured tissue for skin wound healing.

## 5. Conclusions

Overall, these findings indicate that CN triggers c-Src-dependent PKC/ERK phosphorylation to regulate the transcriptional activation of NF-κB and that this signaling pathway governs the cytoskeletal reorganization process to promote stem cell motility. Although our study demonstrated that a treatment of CN onto stem cells leads to significantly enhanced cell migration while also defining the molecular mechanism by which CN acts during this process, further research is required to establish in greater detail the effects of CN on the wound-healing process.

## Figures and Tables

**Table 1:** Scoring of histological changes in wound healing.

Score	Re-Epithelialization
0	Absence of epithelial proliferation in > 70% of the tissue
1	Poor epidermal organization in > 60% of the tissue
2	Incomplete epidermal organization in > 40% of the tissue
3	Moderate epithelial proliferation in > 60% of the tissue
4	Complete epidermal remodeling in > 80% of the tissue

**Table 2:** Properties of nanospheres loaded with curcumin.

	Encapsulation Efficiency (%)	Loading Capacity (%)
CN	87.1 ± 0.1	9.0 ± 0.1

## Abbreviations

Bis I: Bisindolylmaleimide I; CN: Curcumin nanosphere; DLS: Dynamic light scattering; ERK: extracellular signal-regulated kinase; FE-SEM: Field emission scanning edlectron microscope; MAPKs: Mitogen-activated protein kinases; NF-κB: Nuclear factor-kappa B; PFN-1: Profilin-1; PKC: Phosphorylation of protein kinase C; PVDF: Polyvinylidene fluoride; SDS-PAGE: Sodium dodecyl sulfate polyacrylamide gel electrophoresis.
